# Relationship between Vitamin D Insufficiency, Lipid Profile and Atherogenic Indices in Healthy Women Aged 18–50 Years

**DOI:** 10.3390/ejihpe14080155

**Published:** 2024-08-09

**Authors:** Ilham Lhilali, Noura Zouine, Lode Godderis, Adil El Midaoui, Samir El Jaafari, Younes Filali-Zegzouti

**Affiliations:** 1 Cluster of Competence Environment and Health, Faculty of Sciences, Moulay Ismail University, Meknes 50000, Morocco; 2 Higher Institute of Nursing Professions and Health Techniques, Meknes 50000, Morocco; 3 Centre for Health and Environment Unit, Faculty of Medicine, KU Leuven, 3000 Leuven, Belgium; 4 IDEWE, External Service for Prevention and Protection at Work, 3001 Heverlee, Belgium; 5 Faculty of Sciences and Techniques Errachidia, Moulay Ismail University of Meknes, Errachidia 52000, Morocco; adil.el.midaoui@umontreal.ca; 6 Department of Pharmacology and Physiology, Faculty of Medicine, University of Montreal, Montreal, QC H3C 3J7, Canada; 7 BASE Laboratory, Faculty of Sciences and Techniques Errachidia, Moulay Ismail University, Meknes 50000, Morocco

**Keywords:** vitamin D, 25-OH vitamin D, dyslipidemia, atherogenic indices

## Abstract

Although vitamin D insufficiency has been correlated with an increased risk of cardiovascular disease (CVD), there are few data on the association between 25-hydroxyvitamin D (25(OH)D) and atherogenic indices predictive of CVD. This study investigated the relationship of vitamin D status with lipid profile and atherogenic indices in adult women in Morocco. Three hundred women aged 18 to 50 years from Meknes were included. Fasting 25(OH)D and lipid concentrations were assayed by a one-step electrochemiluminescence-based immunoassay and an enzymatic method, respectively. Atherogenic indices (atherogenic index of plasma (AIP), atherogenic coefficient (AC), non-HDL cholesterol (non-HDL-C), Castelli risk indices I and II (CRI-I and II), and CHOLIndex (CI)) were calculated using conventional lipid parameters. Logistic regression models and operating characteristic curve (ROC) analysis were used to assess the relationship of the variables and estimate the threshold of 25(OH)D levels associated with high atherogenic indices. 25(OH) D below 20 ng/mL was significantly associated with an enhanced risk of hypertriglyceridemia and elevated values of AIP, AC, non-HDL-C, and CRI-I with an OR (95% CI) of 4.904 (1.856–12.959), 3.637 (2.149–6.158), 3.589 (1.673–7.700), 2.074 (1.215–3.540), and 2.481 (1.481–4.123), respectively. According to the ROC analysis, the likelihood of hypertriglyceridemia and high values of AIP, AC, non-HDL-C, and CRI-I were associated with 25(OH)D thresholds ≤15.15 ng/mL, ≤17.5 ng/mL, ≤19.8 ng/mL, ≤20.1 ng/mL, and ≤19.5 ng/mL, respectively, all *p* < 0.01. Based on the atherogenic indices, this study indicates that vitamin D below 20 ng/mL may increase the risk of cardiovascular disease in adult women. Additional health measures are essential to raise awareness among women and health professionals of preventing and controlling cardiovascular risk factors, particularly among young individuals.

## 1. Introduction

Vitamin D deficiency is a widespread global health issue, regardless of age, gender, and country of origin [1]. It has been observed that low levels of 25(OH)D are associated with numerous potential negative health consequences such as cancers, autoimmune disorders, infectious diseases, type 1 and 2 diabetes, neurological disorders, and cardiovascular disease [1,2,3,4,5,6,7,8]. Several studies have indicated an inverse association between 25(OH)D and biomarkers of cardiovascular risk including an atherogenic lipid profile [9,10,11,12,13,14,15]. The cardiovascular system is a target for vitamin D. VDR and 1-α-hydroxylase are expressed by cardiomyocytes, smooth muscle cells, and vascular endothelial cells, mainly fibroblasts [16]. Local vitamin D activation by the VDR has various potential cardiovascular benefits including reduced renin production [17,18], relaxation of vascular smooth muscle cells, and reduced output of atherosclerosis-forming foam cells [19,20]. Vitamin D can also reduce inflammation, which is integral to the development of cardiovascular disease (CVD) [19,21,22].

Cardiovascular disease is the leading cause of mortality and morbidity worldwide, representing 32% of all deaths in 2019 [23,24]. In Morocco, two out of five deaths are attributable to CVD (38%), making it the leading cause of death nationwide [25]. CVD is also the leading cause of death in women worldwide and was responsible for 35% of all deaths in 2019 [26]. Contrary to popular belief, cardiovascular disease does not just affect older men and women. This type of problem also increasingly affects young women, with other alarming trends such as stagnation in the incidence and mortality of coronary heart disease and the increase in acute myocardial infarction, particularly in younger women (<55 years) [27,28,29]. The national survey (Stepwise) on risk factors (RF) for non-communicable diseases (NCDs) carried out by the Ministry of Health in 2017–2018 showed that 94.3% of Moroccans aged 18 and over have at least one RF for NCDs, and that 4.9% of individuals aged 40 to 69 have a cardiovascular risk ≥ 30% [30].

Dyslipidemia, which refers to an imbalance in the levels of blood lipids including high levels of triglyceride (TG), total cholesterol (TC), low-density lipoprotein cholesterol (LDL-C), and lower levels of high-density lipoprotein cholesterol (HDL-C), has been identified as a significant risk factor for atherosclerosis and cardiovascular disease [31,32]. Several cross-sectional studies have reported an association of vitamin D with lipid levels and CVD risk [33,34,35,36]. Nevertheless, the association between 25-hydroxyvitamin D concentrations and serum lipids is inconsistent in various studies [37,38]. It has been argued that different combinations of these lipid profile parameters such as the Castelli risk index-I (CRI-I), the CRI-II, the atherogenic coefficient (AC), the CHOLIndex and the atherogenic index of plasma (AIP) are diagnostic alternatives for predicting the risk of developing cardiovascular events and identifying high-risk individuals [39,40,41,42,43,44,45,46,47]. A few studies have assessed the relationship between vitamin D and atherogenic biomarkers. Moreover, most of these studies have been conducted on elderly patients, people with diabetes, or patients with metabolic syndrome [48,49].

Despite the increase in cardiovascular disease risk factors among young people in Morocco and the elevated vitamin D deficiency, particularly among adult women under 50 years of age, estimated at 78.8% in 2019 [50], no study has addressed the association between vitamin D and the biomarkers of cardiovascular disease in this population. Thus, this study aimed to assess the relationship between vitamin D insufficiency, lipid profile, and atherogenic indices in apparently healthy women aged 18–50 and to estimate the optimal 25(OH)D threshold for maintaining lipid parameters and atherogenic indices at least at limit average values.

## 2. Materials and Methods

### 2.1. Study Conception and Subjects

This cross-sectional study was performed in an accredited private medical analysis laboratory in Meknes (Morocco). Three hundred healthy women aged 18–50 years meeting the inclusion criteria were recruited between June and November 2022. The sample size was calculated based on national data on hypovitaminosis D in women aged 18–49 years using the Cochrane formula [51]: n0 = z2pq/e2 (Z = 1.96 for 95%; *p* = 78.8% corresponding to the prevalence of hypovitaminosis D reported in the National Nutrition Survey (ENN 2019) [50]; q is 1-p, and e is the accuracy level required (set at 5%). Thus, 256 women were involved to obtain statistically representative data.

**Figure 1:**
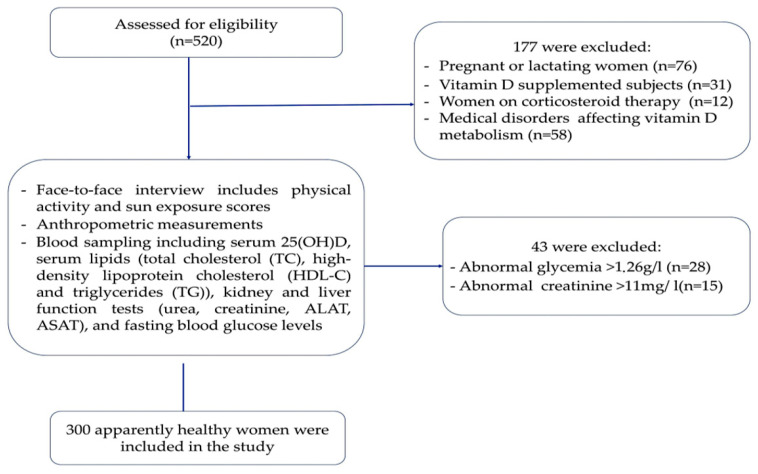
Flowchart of study topics.

Exclusion criteria included women suffering from malabsorption diseases, cancer, liver and kidney disorders, pregnant or lactating women, women who had taken vitamin D supplements in the last six months, and women on drugs known to alter vitamin D metabolism. Women with cardiovascular diseases (e.g., myocardial infarction, stroke…) and diabetes mellitus were also excluded (Figure 1).

Ethical approval (protocol codes CERB-UMI 201901 (January 2019) was received from the Ethics Committee for Biomedical Research of the Moulay Ismail University of Meknes. All women were included after obtaining written informed consent.

### 2.2. Physical Activity and Sun Exposure Scores Assessment

A guided face-to-face questionnaire combining scores and socio-demographic information was completed. Additionally, screening for any diseases that would preclude participation in the study and collecting the participants’ medical history and anthropometric data were undertaken.

#### 2.2.1. Physical Activity (PA) Measurement

The International Physical Activity Questionnaire-Short Version (IPAQ-S) was used to measure the physical activity data. Time spent walking, sitting, and doing moderate- to vigorous-intensity activities per week was calculated. Total physical activity score in METs/min/week was calculated as the sum of (daily minutes of walking × days per week with walking × 3.3 METs), (daily minutes of moderately intense activity × days per week with moderately intense activity × 4 METs), and (daily minutes of vigorous activity × days per week with vigorous activity × 8 METs) [52]. The score was categorized as low intensity (<600 MET-min/week), moderate PA (≥600–2999 METs/min/week), and intense/vigorous PA (at least 3000 MET-min/week) [53].

#### 2.2.2. Sun Exposure Assessment

Sun exposure was assessed using a validated weekly score [54]. The questionnaire comprised two variables: the first covered behaviors influencing sun exposure such as duration, time of day, exposure frequency, exposed body part, and solar protection practices, subdivided into three domains (indoor sun exposure, outdoor sun exposure, and solar protection practices). The second was a two-factor adjustment variable: skin phototype based on Fitzpatrick’s skin type [55] and weather conditions. The total sun exposure score was calculated as the sum of the scores for the three domains × phototype score × outdoor weather conditions score.

### 2.3. Anthropometric and Laboratory Assessment

Anthropometric measurements of height in (cm) and weight in (kg) were determined using a standard procedure to the nearest 0.1 cm and 0.5 kg, respectively. Body mass index (BMI) was calculated as follows: BMI = weight (kg)/height (m)^2^. Obesity was defined as a BMI ≥ 30.0 kg/m^2^, and overweight as a BMI between 25.0 and 29.9 kg/m^2^ [56].

Fasting venous blood of 10 mL was collected. The serum was centrifuged at 2000 rpm for 10 min and analyzed on the day. Serum 25(OH)D concentration was assayed using a one-step delayed chemiluminescent microparticle immunoassay (CMIA) with automated online pre-treatment, total cholesterol and triglyceride were assayed by the standard enzymatic procedure, and HDL-C was analyzed with a direct enzymatic colorimetric test, according to the manufacturer’s instructions. All of the laboratory analyses above were carried out using an Abbott Architect ci 4100 Chemistry Analyzer. The concentration of LDL-C was determined using the Friedewald equation for participants with a TG < 400 mg/dL (LDL-C = TC − (HDL-C + TG/5)) [57].

#### Vitamin D, Lipids Profiles, and Atherogenic Indices Evaluation

According to the Institute of Medicine (IOM) criteria, vitamin D deficiency is defined as 25(OH)D < 12 ng/mL, insufficiency as 25(OH)D between 12 ng/mL and 20 ng, and adequacy as 25(OH)D >20 ng [58,59].

The participants’ lipid profiles were determined based on NCEP ATP III (2001) classification and V Brazilian Guidelines on Dyslipidemia and Prevention of Atherosclerosis [60,61]. The threshold for each dyslipidemia was as follows: TC concentration ≥ 200 mg/dL; LDL-C ≥ 130 mg/dL; TG > 150 mg/dL; and HDL-C < 50 mg/dL.

The atherogenic indices were calculated using the formula described in previous studies [39,41,42]:

AIP = Log (TG/HDL-C)

Non-HDL-C = TC − HDL-C

AC = (TC − HDL-C)/HDL-C

CRI-I = TC/HDL-C

CRI-II = LDL-C/HDL-C

CI = LDL-C − HDL-C (TG < 400 mg/dL)

= LDL-C − HDL-C + 1/5 TG (TG > 400 mg/dL)

AIP > 0.15 was regarded as abnormal in this study [33,36]; non-HDL-C > 130 mg/dL was considered as undesirable [43,62]. Abnormal atherogenic index values predictive of cardiovascular risk are as follows: AC > 3.0, CRI-I > 3.0, and CRI-II > 3.3, and CI > 2.07 [42,62].

### 2.4. Statistical Analysis

Statistical analysis was conducted using R statistical software (version 4.2.1 R Foundation for Statistical Computing). Descriptive data for the participants were presented as medians ± IQR for non-normally continuous variables and expressed as means ± SD for normally continuous variables, determined by a Kolmogorov–Smirnov test. The data at baseline were divided into 25(OH)D levels, and the differences in these variables were tested using Mood’s median test for non-normally distributed variables, the ANOVA test for normally distributed variables, and Pearson’s chi-squared test for categorical variables. The association of lipid profile and atherogenic index with age, BMI, physical activity, sun exposure, and 25(OH)D was analyzed using Spearman’s rank correlation coefficient. The distribution of 25(OH)D concentrations according to the lipid parameters and atherogenic indices was represented using a box plot. A logistic regression analysis model was applied to assess the associations between the abnormal atherogenic index and 25(OH)D level, expressed as odds ratios (OR (95% CI). For multiple regression analysis, vitamin D status was reported as sufficient (>20 ng/mL) and insufficient (<20 ng/mL).

The receiver operating characteristic (ROC) curve and area under the curve (AUC) were used to calculate the accuracy of 25(OH)D in predicting high atherogenic indices. An AUC = 0.5 suggests no discrimination; 0.6 ≥ AUC > 0.5 suggests poor discrimination; 0.7 ≥ AUC > 0.6 indicates acceptable discrimination; 0.8 ≥ AUC > 0.7 indicates excellent discrimination, and an AUC > 0.9 indicates exceptional discrimination [63]. The best cutoff points were the concentration of serum 25(OH)D at the associated criterion of the highest Youden index from the ROC analysis using the MedCalc software (MedCalc^®^ Statistical Software version 22.009 (MedCalc Software Ltd., Ostend, Belgium) [64]. A *p*-value < 0.05 was considered statistically significant.

## 3. Results

### 3.1. Characteristics of Participants According to 25(OH)D Status

In this cross-sectional study of 300 healthy women with a median age of 38, the results showed that 67.6% of participants had a 25(OH)D level below 20 ng/mL, with 34.6% presenting a severe vitamin D deficiency of less than 12 ng/mL, with a median total level of 13.97 ng/mL. Assessment of the lipid profile revealed dyslipidemia in 33.3% of participants. According to the atherogenic index of plasma (AIP), 60.7% of subjects were at high risk of CVD. Abnormal HDL-C, AC, CRI-I, and CRI-II ratios were identified in 43%, 22.3%, 59%, and 6% of participants, respectively (Table 1).

Table 1 shows that the vitamin D-deficient participants were younger (33 vs. 41, *p* = 0.006), more obese (26.2% vs. 6.2%, *p* = 0.001), and had the lowest sun exposure scores (13.21 vs. 18.35, *p* < 0.0001) compared with vitamin D-sufficient status participants.

Concerning the participants’ lipid profile and atherogenic indices according to vitamin D status, the results of Mood’s median test and ANOVA test showed that the TG, AIP, non-HDL-C, AC, CRI I, CRII, and CI levels decreased significantly with improving serum vitamin D status (1.16 vs. 0.81, *p* < 0.0001; 0.30 vs. 0.11, *p* < 0.0001; 1.29 vs. 1.10, *p* = 0.004, 2.35 vs. 1.82, *p* < 0.0001; 3.35 vs. 2.82, *p* < 0.0001; 1.91 vs. 1.63, *p* = 0.035, and 0.52 vs. 0.38, *p* = 0.011, respectively). However, no differences were noted in the TC, HDL, and LDL levels compared to vitamin D status. 

### 3.2. Association of Lipid Profiles and Atherogenic Indices with 25(OH)D Concentrations

The association of the lipid profile and atherogenic indices with age, BMI, physical activity, and sun exposure scores was analyzed using Spearman’s rank correlation coefficient. As shown in Table 2, BMI was positively correlated with the AIP, AC, non-HDL-C, CRI, and CHOLIndex values as well as the TC, LDL-C, and TG serum concentrations. The results of Table 2 revealed that sun exposure and the physical activity scores were positively associated with HDL-C and negatively associated with TG, AIP, AC, CRI-I, and CRI-II. Physical activity was also negatively associated with non-HDL-C and CHOLIndex. However, no association was found between those parameters with age.

**Figure 2:**
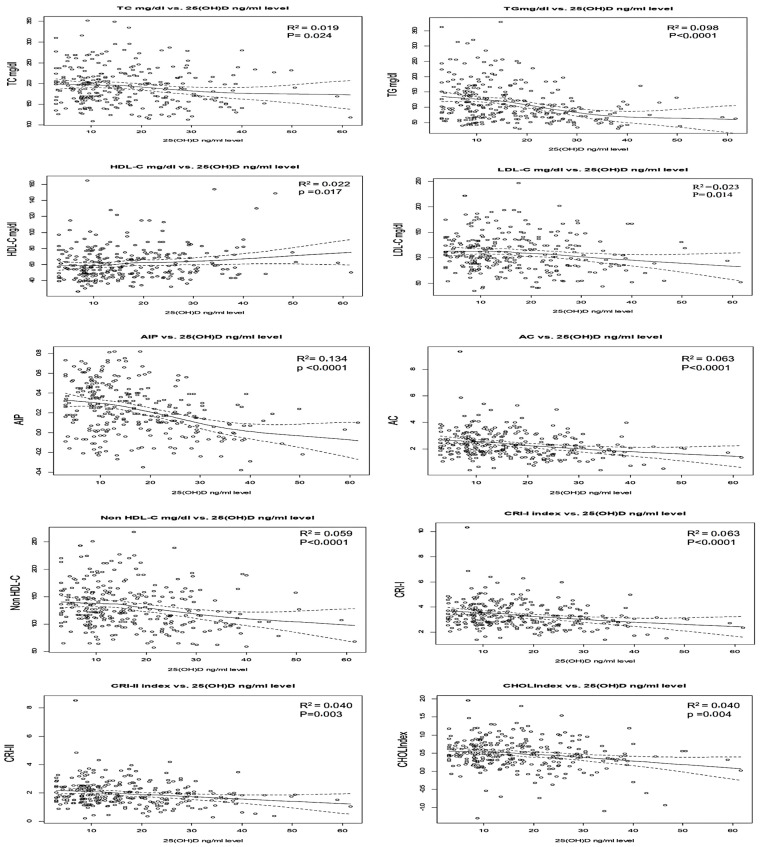
Scatter plot showing the relations of the serum 25(OH)D concentrations with lipid profile and atherogenic indices.

Nonparametric regression analysis was used to evaluate the associations of serum 25(OH)D concentrations with the lipid profile and atherogenic indices. Figure 2 shows that 25(OH)D could account for 13.4% of the variability seen in the AIP, 9.8% of TG, 6.4% of AC, and 6% of non-HDL-C and CRI-I, *p* < 0.0001. Regarding the association with lipid profile, the 25(OH)D serum concentrations were negatively associated with TC, LDL-C, TG, and positively associated with HDL-C.

Table 3 shows that a 1 ng/mL increase in serum 25(OH)D was associated with decreases of 1.53 mg/dL in TG, 0.55 mg/dL in TC, 0.49 mg/dL in LDL-C, and an increase of 0.22 mg/dL in HDL-C. We also found a significantly negative association between 25(OH)D and all atherogenic indices. Each 1 ng/mL increase in 25(OH)D serum was associated with decreases of 0.817 mg/dL in non-HDL-C, 0.009 in AIP, 0.021 in AC and CRI-I, 0.012 in CRI-II, and 0.006 in CHOLIndex, respectively.

**Figure 3:**
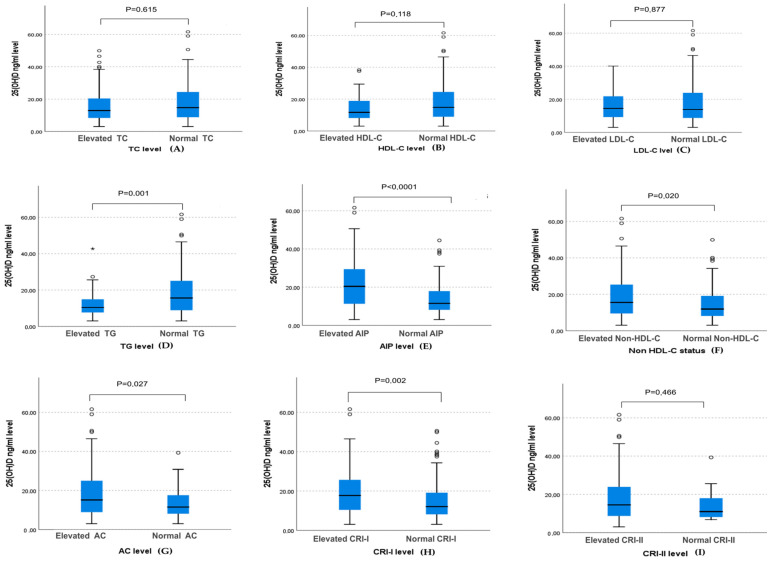
Box plot representing the 25(OH)D concentration according to the lipid profile and atherogenic indices levels. Cutoff values to define dyslipidemia were as follows: TC ≥ 200 mg/dL, TG > 150 mg/dL, LDL-C ≥ 130 mg/dL, and HDL-C < 50 mg/dL. Elevated atherogenic indices were defined as AIP > 0.15; non-HDL-C > 130 mg/dL, AC > 3.0, CRI-I > 3.0, and CRI-II > 3.3. Data are shown as the median. *p*-values were obtained from the Mann–Whitney U test. *p*-values < 0.05 were considered significant.

Figure 3 illustrates that participants with high levels of TG, AIP, AC, non-HDL-C, and CRI I had markedly reduced levels of 25(OH)D concentration compared with those in the normal group (10.40 vs. 15.6, *p* = 0.001; 11.5 vs. 20.4, *p* < 0.0001; 11.5 vs. 15.15, *p* = 0.027; 11.89 vs. 15.5, *p* = 0.020; 12.02 vs. 17.7, *p* = 0.002, respectively). However, there was no significant association between the 25(OH)D serum and elevated TC, HDL-C, LDL-C, and CRI-II levels.

Based on the association results in Table 2 and the box plot (Figure 3), only significant associations with beta > 0.20 were included in the logistic regression and ROC curve analysis. The odds ratio was adjusted to the BMI, physical activity, and sun exposure scores.

### 3.3. The Association between 25(OH)D Level and the Occurrences of Hypertriglyceridemia and High Atherogenic Indices

To assess whether the 25(OH)D level relationship with hypertriglyceridemia and high atherogenic index values differed by vitamin D status, we categorized the participants into two subgroups: vitamin D insufficiency and vitamin D sufficiency.

Binary logistic regression adjusted for the BMI, physical activity, and sun exposure score showed that increasing the serum 25(OH)D concentration significantly reduced the probability of having an elevated level of triglycerides, AIP, AC, non-HDL-C, and CRI-I with an OR (95% CI) of 0.954 (0.916–0.993), *p* = 0.023, 0.940. (0.915–0.965), *p* < 0.0001, 0.958 (0.922–0.995), *p* = 0.026, 0.962 (0935–0.991), *p* = 0.011 and 0.971 (0.948–0.994), *p* = 0.017, respectively (Table 4).

According to vitamin D status, multinomial logistic regression in Table 4 showed that 25(OH)D below 20 ng/mL was significantly associated with an increased risk of hypertriglyceridemia and elevated indices of AIP, AC, non-HDL-C, and CRI-I with an OR (95% CI) of 4.904 (1.856–12.959), *p* = 0.001, 3.637 (2.149–6.158), *p* < 0.0001, 3.589 (1.673–7.700), *p* = 0.001, 2.074 (1.215–3.540), *p* = 0.007, and 2.481 (1.481–4.123), respectively, after adjusting for BMI (Model 1). The ORs then decreased slightly but remained significant after adjusting for BMI, physical activity, and sun exposure score (Model 2).

**Figure 4:**
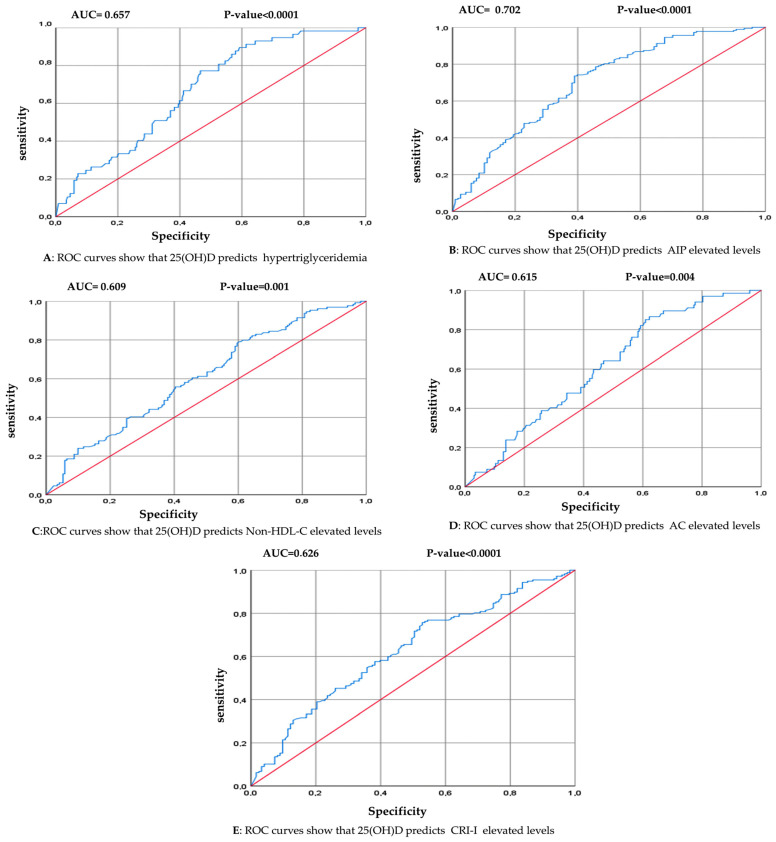
ROC curve analysis estimating the optimal threshold 25(OH)D concentration to predict hypertriglyceridemia and high atherogenic indices. The value of *p* was derived from the ROC analysis. Statistical significance *p* < 0.05. Abbreviations: AUC, area under the curve. (**A**) ROC curve of 25(OH)D and hypertriglyceridemia. (**B**) ROC curve of 25(OH)D and elevated AIP levels. (**C**) ROC curve of 25(OH)D and elevated non-HDL-C levels; (**D**) ROC curve of 25(OH)D and elevated AC levels. (**E**) ROC curve of 25(OH)D and elevated CRI-I levels.

The ROC curve analysis was used to analyze the prognostic accuracy of 25(OH)D and to specify the optimal cutoff predictive of hypertriglyceridemia and high levels of atherogenic indices. The results showed that the area under the curve (AUC) of the association of 25(OH)D with hypertriglyceridemia and the atherogenic indices was significantly acceptable between 60% and 70%, indicating that a decrease in vitamin D concentration could predict increases in TG levels (AUC = 0.669, *p* < 0.0001, cutoff ≤ 15.15 ng/mL), AIP levels (AUC = 0.702, *p* < 0.0001, cutoff ≤ 17.5 ng/mL), AC levels (AUC = 0.615, *p* = 0.004, cutoff ≤ 19.8 ng/mL), non-HDL-C levels (AUC = 0.609, *p* = 0.001, cutoff ≤ 20.1 ng/mL), and CRI-I levels (AUC = 0.626, *p* < 0.0001 cutoff ≤ 19.5 ng/mL). The results of the ROC curve are reported in Table 5 and Figure 4.

## 4. Discussion

Vitamin D deficiency and insufficiency is an epidemic health problem worldwide. This study showed that 67.6% of women aged 18–50 suffered from vitamin D insufficiency below 20 ng/mL. This prevalence is roughly in line with that reported in other studies. In Morocco, the national nutrition survey [50] revealed vitamin D insufficiency in 78.8% of women aged 18 to 49. In the United Arab Emirates, a study of migrant women from Arab and South Asian countries aged 18 and over revealed an overall prevalence of vitamin D deficiency < 20 ng/mL of 67% [65]. In a cross-sectional study in China, Wang et al. [33] reported a vitamin D deficiency in 63.7% of adult women. The causes of vitamin D deficiency in adult women could be explained by conservative clothing, a lack of sun exposure, relatively high obesity, and low vitamin D dietary intake [54,66,67,68,69,70].

Hypovitaminosis D has recently been suggested to be linked to the risk of developing several health problems including cardiovascular disease. Crowe et al. [71], in a large prospective UK study of 180,263 patients aged 18 and over with no history of CVD, suggested that circulating 25(OH)D concentrations below 15 ng/mL are associated with an increased risk of CVD. A meta-analysis of 34 publications involving 180,667 participants found an inverse association between the serum 25(OH)D levels and total cardiovascular events, and the pooled RRs per 10-ng/mL increment were 0.90 (95% CI: 0.86, 0.94) for total CVD events [72].

Various explanations for the role of vitamin D in the development of cardiovascular disease have been suggested. Previous studies have supported that vitamin D may reduce the risk of CVD by blocking the renin-angiotensin system [17,18], decreasing the parathyroid hormone levels, reducing inflammation, lowering coagulation, subsequently reducing atherosclerosis, and increasing insulin production [19,21,22]. Vitamin D may also prevent cardiovascular disease by regulating several genes involved in cell differentiation, proliferation, apoptosis, and angiogenesis [73]. A further possible explanation for the protective effect of vitamin D on cardiovascular disease is the control of lipid parameters [11,12,33,74,75,76,77].

In the present study, lipid profile assessment revealed dyslipidemia in 33.3% of all participants. Nonparametric regression showed that the 25(OH)D concentration was inversely associated with the TC, TG, and LDL-C levels and positively associated with HDL-C. A total of 1 ng/mL in 25(OH)D serum was associated with decreases of 1.53 mg/dL in TG, 0.55 mg/dL in TC, 0.49 mg/dL in LDL-C, and an increase of 0.22 mg/L in HDL-C. These results are consistent with previous research on the association between 25(OH)D and the lipid parameters. In southern Thailand, Jeenduang and Sangkaew [35] indicated that serum 25(OH)D was negatively correlated with the TC, TG, and LDL-C levels in women. Wang et al. [33], in a study of 646 Chinese adult women, showed that the serum 25(OH)D levels were inversely associated with LDL-C and positively associated with TC after adjustment for age and BMI. However, there was no significant association with HDL-C and TG. The authors reported that for every 10 nmol/L increase in 25(OH)D concentration, LDL-C decreased by 0.25 mmol/L and TC increased by 0.39 mmol/L in women [33]. In the Very Large Database of Lipids (VLDL), which included 20,360 adults (≥18 years of age) in the United States, Lupton et al. [78] showed that a serum 25(OH)D level below 20 ng/mL was linked to a significantly decreased serum HDL-C level (−5.1%) and increased TC (+9.4%), LDL-C (+13.5%), and TG (+26.4%) levels compared to the sufficient group.

Vitamin D insufficiency was associated with triglyceride dyslipidemia, with an OR (95% CI) adjusted for BMI, physical activity, and sun exposure of 3.876 (1.427–10.525), *p* = 0.008. In contrast, there was no significant association of vitamin D status with TC, LDL, and HDL dyslipidemia. These results are consistent with those of Alquaiz et al. [11] in their study of apparently healthy men and women, which showed a significant excess in odds ratio for elevated TG levels in association with 25(OH)D deficiency in women (AOR = 3.0; 95% CI = 1.1, 7.9), but not in men. Similarly, Jeenduang and Sangkaew [35] showed that elevated serum 25(OH)D levels were associated with a reduced risk of hypertriglyceridemia and reduced HDL-C levels, particularly in women. Furthermore, our study revealed that a serum vitamin D level ≤ 15.15 ng/mL may predict the likelihood of hypertriglyceridemia with a sensitivity of 77.19% and a specificity of 52.26%.

The pathophysiology of vitamin D deficiency leading to lipid profile is unclear. Multiple mechanisms could explain the relationship between vitamin D and lipid and lipoprotein concentrations. Previous data suggest that increased intestinal calcium absorption may reduce fatty acids in the gut due to the formation of insoluble calcium fatty acid soap complexes, increase fat absorption, and reduce the hepatic triglyceride levels [79]. Serum LDL-C levels are thought to be reduced by decreased fat absorption, particularly of saturated fatty acids [80,81]. Moreover, calcium may favor the translation of cholesterol into bile acids, thus reducing cholesterol levels [82]. Vitamin D has also been shown to play a role in cholesterol transport by regulating the apolipoprotein A-1 levels, and may act to reduce the LDL-C uptake, decrease foam cell formation, and increase HDL-C production [83,84,85]. In fact, earlier studies have provided robust evidence that vitamin D deficiency may be linked to impaired B-cell function and insulin resistance. These conditions may affect lipoprotein metabolism and ultimately lead to elevated TG levels and reduced HDL-C levels [86,87]. Similarly, vitamin D can affect lipoprotein metabolism, with Larrick et al. [88] demonstrating that by regulating the expression of genes involving lipid metabolism, vitamin D enhances the HDL-C levels, decreases fatty acid synthesis, and improves fatty acid β-oxidation, thereby lowering the triglyceride levels.

An alternative mechanism of this association may be related to confounding factors affecting both 25(OH)D and the lipid levels such as obesity, lower amounts of physical activity, and sun exposure. Our results showed that vitamin D deficiency and insufficiency was associated with high BMI and low physical activity and sun exposure scores. These results are similar to other studies [66,89,90,91,92,93]. In addition, this study showed that BMI has a positively significant association with TC, TG and LDL-C, which could increase the risk of dyslipidemia and obesity-related cardiovascular events [94,95,96]. In Morocco, there has been an alarming increase in the number of overweight and obese women (29.2% and 28.4%, respectively) [50]. Associated with these changes in BMI, other pathologies may emerge such as metabolic syndrome and diabetes, which increase the risk of developing CVD. Excess adipose tissue appears to be directly responsible for dyslipidemia associated with abdominal obesity by inducing an increase in the flow of free fatty acids to the liver and contributing to insulin resistance [97].

Moreover, the sun exposure and physical activity scores were found to be positively associated with HDL-C and negatively associated with TG levels. Weller [98] demonstrated that exposure to sunlight has health benefits independent of vitamin D. The skin contains significant stores of nitrogen oxides, which can be converted to NO by UV radiation and exported to the systemic circulation, thus causing arterial vasodilation and decreased blood pressure. Other studies suggest that enhancing the bioavailability of nitric oxide could have beneficial effects on obesity and the lowering of triglyceride levels [99,100]. Additionally, regular physical activity and fitness have shown inverse associations with cardiovascular disease in many observational studies [101,102,103]. It has been shown that physical activity can lead to significant positive changes in lipid profile [104,105].

Other than these risk factors, the identification and validity of new measures of cardiovascular disease risk have attracted considerable interest of late. In fact, atherogenic indices including AIP, AC, CRI-I, non-HDL-C and others have been proposed as alternative biomarkers to diagnose individuals at CVD risk beyond the usual risk factors [40,45,106]. The AIP was suggested by the National Cholesterol Education Program [60] as a powerful predictor of atherosclerosis, and coronary heart events could be used as an effective mass screening method to detect patients at high risk of cardiovascular disease [106,107]. Non-HDL-C was recommended by the 2019 ESC/EAS guidelines for the management of dyslipidemia as an effective biomarker for the estimation of cardiovascular risk in individuals with elevated TG levels, T2DM, obesity or a very high risk of cardiovascular events, and low LDL levels [108]. For CRI-I, it has been particularly shown to reflect coronary plaque formation and intima-media thickness in the carotid arteries of young adults [39].

The current study showed elevated cardiovascular risk according to atherogenic index values, in particular AIP, CRI-I, non-HDL-C, and AC (60.7%, 59%, 43%, and 22.3% respectively). Molani et al. [62] reported that the prevalence of elevated AIP, AC, CI, CRI-I, CRI-II and non-HDL-C indices in adult healthy women were 64.5%, 36.2%, 20.4%, 77%, 7.2%, and 44.7%, respectively. According to the AIP levels, Fernández-Macías et al. [46] showed that around 55% of healthy Mexican women aged 18 and over were at moderate or high risk of cardiovascular disease. Wang et al. found a lower incidence among Chinese women, at 25% [33]. Thus, atherosclerosis may begin early in life and go undetected for a prolonged period before developing into an advanced, clinically presentable phase [109,110].

The 25(OH)D values were negatively and independently correlated with the AIP, AC, non-HDL-C, and CRI-I levels. Logistic regression showed that vitamin D insufficiency compared with the sufficient group significantly increased the risk of elevated AIP, AC, non-HDL-C, and CRI-I values with an OR (95% CI) of 3.637 (2.149–6.158), *p* < 0.0001; 3.589 (1.673–7.700), *p* = 0.001; 2.074 (1.215–3.540), *p* = 0.007; and 2.481 (1.481–4.123), respectively, after adjustment for BMI. These findings corroborate those presented in other studies. Lupton et al. [78] showed that serum 25(OH)D deficiency (<20 ng/mL) was associated with higher levels of non-HDL-C (+15.4%, +20.3 mg/dL). Barbalho et al. reported that patients with impaired vitamin D values had significantly higher values of CRI-I, CRI-II, and non-HDL-C [8]. Similarly, Pokhrel et al. [111] demonstrated that 25(OH)D was negatively associated with AIP, AC, and non-HDL-C. In addition, in a longitudinal community study of 13,039 participants in the ARIC (Atherosclerosis Risk in Communities) study, Faridi et al. [112] reported that deficient 25(OH)D was prospectively associated with greater CRI-I ratio after considering factors such as diabetes and adiposity.

A gender difference was reported by Wang et al., who showed that vitamin D levels were inversely significantly associated with AIP levels in men, but not in women [33]. Huang et al. confirmed a relatively stronger association of AIP and vitamin D in men [36], while Izadi et al. showed a significant negative association of vitamin D and AIP in both sexes [113]. In a case–control study among participants with T2DM, Mahmoodi et al. [49] reported that all atherogenic indices including AIP, CRII, and AC decreased significantly with improving vitamin D status in men in the control group only. Furthermore, studies in children and young adolescents revealed an inverse association between the vitamin D levels and atherogenic indices [114,115,116,117]. Thus, maintaining adequate vitamin D status contributes to the early prevention of cardiovascular disease in individuals with or without risk factors.

ROC curve analysis showed that a decrease in vitamin D concentration independently predicted an increase in the atherogenic index values. Thresholds predicting elevated AIP, AC, non-HDL-C, and CRI-I values were serum 25(OH)D ≤ 17.5 ng/mL, ≤19.8 ng/mL, ≤20.1 ng/mL, and ≤19.5 ng/mL respectively, all *p* < 0.001. These results confirm that serum 25(OH)D concentrations below 20 ng/mL imply an increased risk of short- or long-term cardiovascular disease. Wang et al. [43] reported a strong association between low 25(OH)D concentration and the total risk of cardiovascular events: RR = 1.52 (1.30–1.77), cardiovascular mortality: RR = 1.42 (1.17–1.71), coronary heart disease: RR = 1.38 (1.21–1.57). The risk appeared when the serum 25(OH)D concentration was below 60 nmol/L (24 ng/mL) and increased when the concentration decreased from 60 to 20 nmol/L (24 to 8 ng/mL). These associations remained (or were even more significant) after adjustment for cardiovascular risk factors known to influence vitamin D insufficiency (obesity, low outdoor activity, ethnicity). In contrast, Barbalho et al. [8] indicated that for a subject to maintain triglycerides, CRI-I, CRI-II, and non-HDL-C metabolic parameters, at least at borderline values, the levels of VD should be 37.64, which is above the reference values, implying that further in-depth studies are needed to determine the 25(OH)D threshold value predictive of dyslipidemia and high levels of atherogenic indices.

Our study has certain limitations. First, the generalizability of the results of our study to the general population is restricted; the study sample was based only on women from the city of Meknes. Due to the cross-sectional nature of this study, the results should not be considered in terms of cause-and-effect relationships. Another limitation is the absence of baseline data affecting the lipid and vitamin D status such as dietary intake, which can provide information on the participants’ daily lipid and vitamin D intake. Finally, although the sample size was sufficient to achieve the required significance, a larger sample could have strengthened the results.

Despite these limitations, the study is well-founded. This is the first study to examine healthy adult women and evaluate the association of vitamin D with lipid and atherogenic profiles in Morocco. This could open up new avenues of research and diagnosis in the scientific and medical fields.

## 5. Conclusions

Vitamin D deficiency and insufficiency are common among adult women in Morocco. Moreover, there is increasing evidence that vitamin D is a non-classical risk factor for CVD that may influence or aggravate other risk factors such as hypertension, dyslipidemia, insulin resistance, and obesity. Our results suggest that serum 25(OH)D levels below 20 ng/mL may be correlated with elevated triglyceride levels and atherogenic indices, particularly those known to be predictors of premature atherosclerosis and coronary events. Randomized controlled trials (RCTs) are needed to determine the adequate vitamin D level that reduces the risk of cardiometabolic diseases. Therefore, additional efforts are needed to raise awareness among women and healthcare providers about preventing and controlling metabolic and cardiovascular risk factors, especially young individuals.

## Figures and Tables

**Table 1:** Clinical and biological characteristics of participants stratified by 25(OH)D status.

Participants Characteristics	Total = 300	25(OH)D <12 ng/mL N = 129	25(OH)D (12–20 ng/mL) N = 73	25(OH)D >20 ng/mL N = 97	*p*-Value
**Clinical characteristics**	**N (%)**				
Age (years) *	38 ± 18 (18–50)	33 ± 20 (20–50)	38 ± 18 (18–50)	41 ± 14 (19–50)	**0.006 ^b^**
**18–28**	96 (32)	53 (40.8)	23 (31.5)	20 (20.6)	**0.020 ^c^**
**29–39**	89 (29.7)	37 (28.5)	22 (30.1)	30 (30.9)	
**40–50**	115 (38.3)	40 (30.8)	28 (38.4)	47 (48.5)	
BMI (kg/m^2^) *	25.74 ± 4.81 (14.53–38.10)	26.45 ± 5.92 (17.47–38.10)	26.35 ± 4.60 (14.53–36.65)	24.84 ± 4.08 (14.53–32.39)	**0.008 ^b^**
**Normal weight**	122 (40.7)	45 (34.6)	25 (34.2)	52 (53.6)	0.001 ^c^
**Overweight**	124 (14.3)	51 (39.2)	34 (46.6)	39 (40.2)	
**Obese**	54 (18)	34 (26.2)	14 (19.2)	6 (6.2)	
Sun exposure score *	15.25 ± 6.86	13.21 ± 5.75 (5.06 -23.06)	14.75 ± 8.09 (7.5–31)	18.35 ± 8.99 (8.5–34.50)	**<0.0001 ^b^**
Physical activity score (*MET*-min/week) *	2038.8 ± 1838.7 (519–4458)	1778.4 ± 1721.1 (519.6–4458)	1597.6 ± 1318.8 (519–4197.8)	2896.6 ±1300.3 (799–4197)	<0.0001 ^b^
**low physical activity score** **<600 MET-min/week**	19 (6.3)	9 (6.9)	10 (13.7)	0 (0)	**<0.0001 ^c^**
**Moderate physical activity score ≥600–2999 METs/min/week**	213 (71)	97 (74.6)	54 (74)	62 (63.9)	
**High physical activity score** **≥3000 MET-min/week**	68 (22.7)	24 (18.5)	09 (12.3)	35 (36.1)	
**Biochemical Characteristics**	**N (%)**				
25(OH)D (ng/mL) *	13.97 ± 15.17 (3–61.60)	8.30 ± 3.43 (3–11.89)	15.70 ± 4.04 (1.02–19.80)	27.84 ± 10.52 (20.10–61.60)	**<0.0001 ^b^**
TC (mg/dL) *	188 ± 55 (109–352)	195 ± 49 (109–352)	190 ± 65 (109–349)	177 ± 48 (113–287)	0.153 ^b^
**Elevated TC (mg/dL)**	91 (30.3)	41 (31.5)	26 (35.6)	24 (24.7)	0.288 ^c^
HDL-C (mg/dL) *	56 ± 23 (0.26–165)	55 ± 24 (26–165)	56 ± 21 32–128)	60 ± 24 (32–154)	0.153 ^b^
**Low HDL-C (mg/dL)**	56 (18.7)	29 (22.3)	15 (20.5)	12 (12.4)	0.147 ^c^
LDL-C (mg/dL) *	104 ± 044 (35–247)	107 ± 36 (35–222)	107 ± 48 (58–247)	96 ± 44 (41–202)	0.334 ^b^
**Elevated LDL-C (mg/dL)**	50 (16.7)	21 (16.2)	15 (20.5)	14 (14.4)	0.559 ^c^
TG (mg/dL) *	97 ± 75 (29–379)	116 ± 86 (40–320)	109 ± 87 (29–379)	81 ± 42 (30–227)	**<0.0001 ^b^**
**Elevated TG (mg/dL)**	57 (19%)	33 (25.4)	19 (26)	5 (5.2)	**<0.0001 ^c^**
**Atherogenic Indices Characteristics**	**N (%)**				
**AIP ^(N”)^**	0.23 ± 0.25 (−0.38–0.82)	0.30 ± 0.25 (−0.23–0.81)	0.28 ± 0.25 (−0.35–0.82)	0.11 ± 0.25 (−0.38–0.58)	**<0.0001 ^a^**
**Elevated AIP**	182 (60.7)	96 (73.8)	50 (68.5)	36 (37.1)	**<0.0001 ^c^**
Non-HDL-C mg/dL *	123 ± 50 (57–268)	129 ± 46 (64–251)	130 ± 50 (72–268)	110 ± 50 (57–239)	**0.004 ^b^**
**Elevated Non-HDL-C**	129 (43)	65 (50)	36 (49.3)	28 (28.9)	**0.003 ^c^**
AC *	2.19 ±1.17 (0.39–9.35)	2.35 ± 1.29 (0.39–9.35)	2.32 ± 1.32 (0.56–5.29)	1.82 ± 0.91 (0.40–4.98)	**<0.0001 ^b^**
**Elevated AC**	67 (22.3)	35 (26.9)	23 (31.5)	9 (9.3)	**0.001 ^c^**
CRI-I *	3.19 ±1.17 (1.39–10.35)	3.35 ± 1.29 (1.39–10.35)	3.30 ± 1.33 (1.56–6.29)	2.82 ± 0.91 (1.40–5.98)	**<0.0001 ^b^**
**Elevated CRI-I**	177 (59)	88 (67.7)	48 (65.8)	41 (42.3)	**<0.0001 ^c^**
CRI-II *	1.79 ±0.98 (0.21–8.54)	1.91 ± 0.99 (0.21–8.54)	1.98 ± 1.05 (0.45–3.97)	1.63 ± 0.81 (0.29–4.21)	**0.035 ^b^**
**Elevated CRI-II**	18 (6)	10 (7.7)	5 (6.8)	3 (3.4)	0.332 ^c^
CHOLIndex (CI) *	0.45 ± 0.44 (−1.30–1.96)	0.52 ± 0.43 (−1.30–1.96)	0.53 ± 0.47 (−0.70–1.80)	0.38 ±0.41 (−1.10–1.54)	**0.011 ^b^**
**Elevated CI**	0%		-	-	-

**Abbreviations:** 25(OH)D, 25-hydroxyvitamin D; BMI, body mass index; TC, total cholesterol; TG, triglycerides; LDL-C, low-density lipoprotein; HDL-C, high-density lipoprotein; AIP, atherogenic index of plasma, Non-HDL-C, non-HDL-cholesterol; CRI, Castelli’s risk index I, CRI II, Castelli risk index-II; AC, atherogenic coefficient, CI, CHOLIndex. **Note:** Data presented as N (%) for categorical variables, mean ± SD (Min–Max) for continuous normal variables (N”), and medians ± IQR (Min–Max) for continuous non-normal variables (*). The difference between variables and 25(OH)D level was calculated by the Univariate ANOVA test for normally distributed variables (a), Mood’s median test for non-normally distributed continuous variables (b), and Pearson’s chi-squared test for the categorical variables (c). A *p* < 0.05 was considered significant.

**Table 2:** Correlation of participant characteristics with lipid profiles and atherogenic indices.

	Age (year)	BMI (kg/m^2^)	Sun Exposure Score	Physical Activity Score (MET-min/week)
**TC (mg/dL)**	0.085	0.288 ***	−0.027	−0.072
**HDL-C (mg/dL)**	0.054	0.071	0.204 ***	0.226 ***
**LDL-C (mg/dL)**	0.106	0.196 **	−0.034	−0.060
**TG (mg/dL)**	−0.50	0.266 ***	−0.232 ***	−0.371 ***
**AIP**	−0.052	0.226 ***	−0.306 ***	−0.432 ***
**AC**	0.032	0.141 *	−0.206 ***	−0.264 ***
**Non-HDL-C (mg/dL)**	0.060	0253 ***	−0.091	−0.147 *
**CRI-I**	0.032	0.141 *	−0.206 ***	−0.264 ***
**CRI-II**	0.078	0.096	−0.157 **	−0.194 **
**CHOLIndex**	0.091	0.136 *	−0.106	−0.133 *

Spearman test showing relationships of lipid profile and atherogenic indices, with serum 25(OH)D level, age, BMI, sun exposure score, and physical activity score. Signification codes: 0 < *** < 0.001 < ** < 0.01 < * < 0.05

**Table 3:** Nonparametric linear regression of lipid profile and atherogenic indices with 25(OH)D concentrations.

Dependent Variables	25(OH)D ng/mL			
	B	B (95% CI)	Beta	*p*-Value
**TC**	−0.555	−0.701 to −0.414	−0.130	**0.024**
**HDL-C**	0.216	0.159 to 0.272	0.137	**0.017**
**LDL-C**	−0.484	−0.569 to −0.349	−0.144	**0.014**
**TG**	−1.528	−1.688 to −1.362	−0.314	**<0.0001**
**AIP**	−0.009	−0.011 to −0.006	−0.366	**<0.0001**
**AC**	−0.021	−0.024 to −0.018	−0.261	**<0.0001**
**Non-HDL-C**	−0.817	−0.930 to −0.707	−0.231	**<0.0001**
**CRI-I**	−0.021	−0.024 to −0.018	−0.261	**<0.0001**
**CRI-II**	−0.012	−0.014 to −0.01	−0.176	**0.003**
**CHOLIndex**	−0.006	−0.007 to −0.005	−0.169	**0.004**

Β coefficient is a standardized coefficient in nonparametric regression analysis. Beta corresponds to the Rho Spearman coefficient.

**Table 4:** Logistic regression for the association between atherogenic indices.

Dependent Variables ^a^		25(OH)D ng/mL				25(OH)D Status ^b^			
		B	Wald	OR (95% CI)	*p*-Value	B	Wald	OR (95% CI)	*p*-Value
**TG**	Unadjusted	−0.071	13.490	0.931 (0.897–0.967)	**<0.0001**	1.846	14.40	6.336 (2.442–16.443)	**<0.0001**
	Model 1	−0.056	8.086	0.946 (0.910–0.983)	**0.004**	1.590	10.28	4.904 (1.856–12.959)	**0.001**
	Model 2	−0.047	5.164	0.954 (0.916–0.993)	**0.023**	1.355	7.065	3.876 (1.427–10.525)	**0.008**
**AIP**	Unadjusted	−0.078	34.926	0.924 (0.901–0.949)	**<0.0001**	1.468	31.427	4.340 (2.598–7.251)	**<0.0001**
	Model 1	−0.072	28.495	0.931 (0.906–0.956)	**<0.0001**	1.291	23.111	3.637 (2.149–6.158)	**<0.0001**
	Model 2	−0.062	20.811	0.940 (0.915–0.965)	**<0.0001**	1.069	14.642	2.912 (1.684–5.035)	**<0.0001**
**AC**	Unadjusted	−0.050	9.753	0.951 (0.921–0.981)	**0.001**	1.364	12.687	3.911 (1.847–8.284)	**<0.0001**
	Model 1	−0.047	7.885	0.954 (0.924–0.986)	**0.005**	1.278	10.765	3.589 (1.673–7.700)	**0.001**
	Model 2	−0.042	4.924	0.958 (0.922–0.995)	**0.026**	1.103	7.511	3.190 (1.463–6.957)	**0.006**
**Non-HDL-C**	Unadjusted	−0.041	11.325	0.959 (0.937–0.983)	**0.001**	0.892	11.382	2.440 (1.453–4.097)	**0.001**
	Model 1	−0.32	6.381	0.969 (0.945–0.993)	**0.012**	0.729	7.154	2.074 (1.215–3.540)	**0.007**
	Model 2	−0.038	6.497	0.962 (0935–0.991)	**0.011**	0.716	6.318	2.047 (1.171–3.578)	**0.012**
**CRI-I**	Unadjusted	−0.039	12.10	0.961 (0.939–0.982)	**<0.0001**	1.020	16.116	2.772 (1.685–4.561)	**<0.0001**
	Model 1	−0.034	8.264	0.967 (0.944–0.989)	**0.004**	0.905	11.998	2.481 (1.481–4.123)	**0.001**
	Model 2	−0.029	5.727	0.971 (0.948–0.994)	**0.017**	0.823	9.098	2.278 (1.334–3.888)	**0.003**

^a^ Dependent variables are the elevated level of each constant; ^b^ Vitamin D status was categorized into 25(OH)D > 20 ng/mL as sufficient, and 25(OH)D < 20 ng/mL as insufficient, vitamin D sufficiency was the reference level. Model 1: Odds ratio adjusted for BMI. Model 2: Odds ratio adjusted for BMI, physical activity, and sun exposure score. Statistical significance: *p* ≤ 0.05.

**Table 5:** ROC curve analysis of 25(OH)D accuracy in predicting high triglycerides and atherogenic indices.

Variable	AUC (95% CI)	*p*-Value	Youden Index	Cutoff (ng/mL)	Sensitivity (%)	Specificity (%)
TG > 150 mg/dL	0.657 (0.586–0.727)	<0.0001	0.294	≤15.15	77.19	52.26
AIP > 0.15	0.702 (0.641–0.764)	<0.0001	0.346	≤17.5	73.63	61.02
AC > 3.0	0.615 (0.545–0.685)	0.004	0.243	≤19.8	86.57	37.77
Non-HDL-C > 130 mg/dL	0.609 (0.545–0.672)	0.001	0.188	≤20.1	79.07	39.77
CRI-I > 3.0	0.626 (0.562–0.690)	<0.0001	0.228	≤19.5	75.71	47.15

AUC, area under the curve. The value of cutoff, AUC, sensitivity, specificity, and Youden index were calculated by ROC analysis. *p* < 0.05 indicates statistical significance.

## Data Availability

The complete datasets developed and analyzed for the current research are available from Lhilali Ilham, on request.
